# The environmental impact of energy consumption and carbon emissions in radiology departments: a systematic review

**DOI:** 10.1186/s41747-024-00424-6

**Published:** 2024-02-29

**Authors:** Andrea Roletto, Moreno Zanardo, Giuseppe Roberto Bonfitto, Diego Catania, Francesco Sardanelli, Simone Zanoni

**Affiliations:** 1 Department of Mechanical and Industrial Engineering, Università degli Studi di Brescia, Via Branze 38, 25123 Brescia, Italy; 2 Radiology Unit, IRCCS Policlinico San Donato, Via Morandi 30, 20097 San Donato Milanese, Italy; 3 Department of Information Engineering, Università degli Studi di Brescia, Via Branze 38, 25123 Brescia, Italy; 4 Health Professions Leadership and Management Unit, IRCCS Ospedale San Raffaele, Via Olgettina 60, 20132 Milan, Italy; 5 Department of Biomedical Sciences for Health, Università degli Studi di Milano, Via Mangiagalli 31, 20133 Milan, Italy; 6 Department of Civil, Environmental, Architectural Engineering and Mathematics, Università degli Studi di Brescia, Via Branze 43, 25123 Brescia, Italy

**Keywords:** Carbon emissions, Electricity, Energy savings, Environmental sustainability, Radiology

## Abstract

**Objectives:**

Energy consumption and carbon emissions from medical equipment like CT/MRI scanners and workstations contribute to the environmental impact of healthcare facilities. The aim of this systematic review was to identify all strategies to reduce energy use and carbon emissions in radiology.

**Methods:**

In June 2023, a systematic review (Medline/Embase/Web of Science) was performed to search original articles on environmental sustainability in radiology. The extracted data include environmental sustainability topics (*e.g.*, energy consumption, carbon footprint) and radiological devices involved. Sustainable actions and environmental impact in radiology settings were analyzed. Study quality was assessed using the QualSyst tool.

**Results:**

From 918 retrieved articles, 16 met the inclusion criteria. Among them, main topics were energy consumption (10/16, 62.5%), life-cycle assessment (4/16, 25.0%), and carbon footprint (2/16, 12.5%). Eleven studies reported that 40–91% of the energy consumed by radiological devices can be defined as “nonproductive” (devices “on” but not working). Turning-off devices during idle periods 9/16 (56.2%) and implementing workflow informatic tools (2/16, 12.5%) were the sustainable actions identified. Energy-saving strategies were reported in 8/16 articles (50%), estimating annual savings of thousand kilowatt-hours (14,180–171,000 kWh). Cost-savings were identified in 7/16 (43.7%) articles, ranging from US $9,225 to 14,328 per device. Study quality was over or equal the 80% of high-quality level in 14/16 (87.5%) articles.

**Conclusion:**

Energy consumption and environmental sustainability in radiology received attention in literature. Sustainable actions include turning-off radiological devices during idle periods, favoring the most energy-efficient imaging devices, and educating radiological staff on energy-saving practices, without compromising service quality.

**Relevance statement:**

A non-negligible number of articles — mainly coming from North America and Europe — highlighted the need for energy-saving strategies, attention to equipment life-cycle assessment, and carbon footprint reduction in radiology, with a potential for cost-saving outcome.

**Key points:**

• Energy consumption and environmental sustainability in radiology received attention in the literature (16 articles published from 2010 to 2023).

• A substantial portion (40–91%) of the energy consumed by radiological devices was classified as “non-productive” (devices “on” but not working).

• Sustainable action such as shutting down devices during idle periods was identified, with potential annual energy savings ranging from 14,180 to 171,000 kWh.

**Graphical Abstract:**

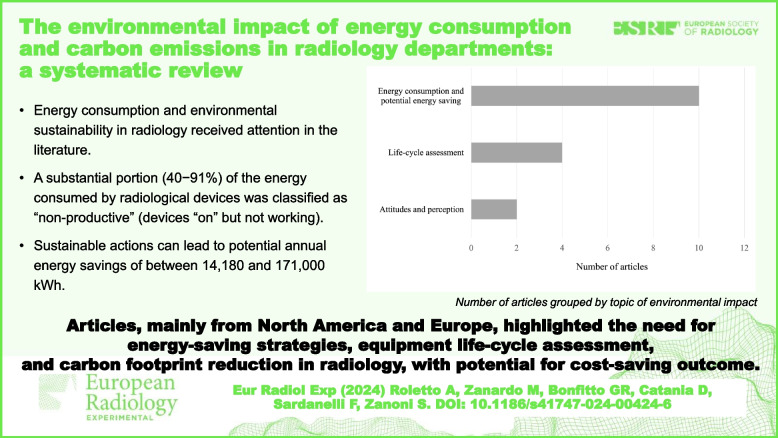

**Supplementary Information:**

The online version contains supplementary material available at 10.1186/s41747-024-00424-6.

## Introduction

In recent years, there has been a growing recognition of the urgent need for environmental sustainability across various sectors of society. As a result, individuals, organizations, and industries are taking proactive steps to minimize their ecological footprint, to ensure not only environmental benefits but also substantial economic savings [1]. Climate change is causing a range of impacts on communities such as more frequent extreme weather, air pollution, changing distribution of infectious diseases, mental health impacts, and others [2].

Within the healthcare sector, environmental sustainability has emerged as a critical concern [3–5]. Healthcare facilities, including radiology departments, have traditionally been associated with high-energy consumption, carbon emissions, waste generation, and the use of potentially harmful substances. However, the realization of the healthcare sector’s substantial contribution to environmental degradation has spurred a transformative shift toward sustainability [6, 7].

In this context, the Greenhouse Gas (GHG) Protocol Corporate Standard [8] establishes standards and guidance for organizations in preparing GHG emissions inventories, outlined by the Kyoto Protocol. Its objectives include ensuring a true and fair representation of emissions, simplifying inventory compilation, providing data for strategic emissions management, facilitating participation in GHG programs, and enhancing consistency and transparency in accounting and reporting across companies and programs.

In addition, considering the categorization of the GHG Protocol Corporate Standard [8] for carbon emissions associated with companies (Scope 1 for “direct GHG emissions”; Scope 2 for “electricity indirect GHG emissions”; and Scope 3 for “other indirect GHG emissions”), health care facilities play a role in all three of these categories.

Radiology departments, crucial in the diagnosis and treatment of various medical conditions, have a particular responsibility to align their practices with environmental sustainability principles. Studies show that diagnostic departments account for about 9% of the carbon emission footprint in medicine [9, 10]. The health sector is responsible for a part of the global greenhouse gas emissions of about 4–10% [10–12]. Other studies have shown that computed tomography (CT) and magnetic resonance imaging (MRI) equipment account for a large portion of the hospital’s total energy consumption and carbon emissions (Scope 2) [10, 13–17]. In radiology departments, the main contributor to climate change is high electricity consumption caused by using energy-intensive medical devices such as CT scanners, MRI systems, and workstations [16, 17]. Additionally, a significant portion of medical equipment remains on and nonproductive for nearly one-third of the day [14]. Furthermore, the cooling processes of radiology equipment and the energy consumption generated by the enterprise picture archiving and communication system (PACS) contribute significantly to the energy consumption and carbon emission (Scope 1 and Scope 2) for hospitals, especially when these systems are left on overnight, as commonly practiced in radiology departments [16].

In an intermediate-size or large radiology department, a considerable number of workstations always remain in operation, regardless of their actual use. This prevalent practice is particularly evident in hospital facilities, where hundreds of workstations contribute significantly to unnecessary energy consumption. As a result, excessive energy waste exacerbates the environmental footprint associated with radiology [18]. For those reasons, hospital radiology departments have great potential in reducing energy consumption, which is accessible by developing workflow optimization processes, using control systems, and improving and optimizing the utilization of used equipment of imaging departments.

To our knowledge, the only systematic reviews published so far in the radiological area have focused on interventional radiology [19] and radiation oncology [20]. Therefore, the purpose of this systematic review was to identify all strategies and approaches to minimize energy use and carbon emissions in radiology departments.

## Materials and methods

No ethics committee approval was needed to perform this systematic review, and it was reported according to the Preferred Reporting Items for Systematic Reviews and Meta-Analyses (PRISMA) statement [21].

### Systematic search

In June 2023, a systematic search was conducted on MEDLINE (PubMed), Embase (Elsevier), and Web of Science to find original articles on environmental sustainability in radiology department. The search was limited to original studies written in English, published on peer-reviewed journals, with an available abstract. The search strings were built using the following strategy, based on the Population, Intervention, Comparison, and Outcome (PICO) model and adjusted for each database considering their own unique indexing systems, terminology, and search algorithms:

- 'Environmental sustainability'/exp + synonyms
- 'Radiology'/exp + synonyms OR 'radiology department'/exp
- 'Energy consumption'/exp + synonyms OR 'carbon emission'/exp OR 'recycling'/exp OR 'waste'/exp”

The full search strings were reported in Supplementary file 1.

Three researchers (A.R., G.R.B., and M.Z., with 1 to 7 years of experience in diagnostic imaging research) performed in consensus an initial screening of the retrieved articles, excluding reviews, case reports, and studies that only comprised automatic computer analyses. The full text was downloaded for all studies included at this first selection, and a second screening was performed. Finally, references of included articles and reviews were manually searched to check for further eligible studies.

We included a study if it was as follows: (1) focused on environmental sustainability strategies and (2) had sustainability endpoint in diagnostic radiology departments, radiology imaging services, or in diagnostic radiology medical school. We excluded the following: (1) studies focused on hospitals in general or in setting different from diagnostic radiology department; (2) reviews, editorials, and viewpoints; and (3) abstracts or conference proceedings.

### Data extraction

For all articles included at the final selection, the same researchers who selected articles performed independently data extraction. In case of disagreement among readers, arbitration was performed in consensus. For each study, the extracted data (when present) were as follows: year of publication; continent and country of publication; study design (prospective or retrospective); environmental sustainability topic (energy consumption, carbon footprint, life-cycle assessment); type of carbon emission or energy consumption (direct carbon emission/energy consumption, for diagnostic radiology activity delivery, or indirect carbon emission/energy consumption, from sources such as patients, students and workers travel, and resource life cycle); types of radiological devices involved; unit of measurements system; data collection method; and sustainable actions described. Findings from the included studies that reported on environmental sustainability in the radiology setting are presented through a formal narrative synthesis.

### Study quality appraisal

One researcher (A.R.) with 1 year of experience in diagnostic imaging research reviewed the quality of the included articles, using the QualSyst tool [22], using the checklist for qualitative studies.

## Results

### Study selection and methodological quality assessment

Starting from 400 records identified through search query on MEDLINE, 823 on Embase, and 474 on Web of Science and 8 identified through other sources such as references from included works, 918 were identified after duplicates removed. A total of 858 articles were excluded from title and abstract, while the remaining 60 were downloaded for individual assessment.

**Fig. 1:**
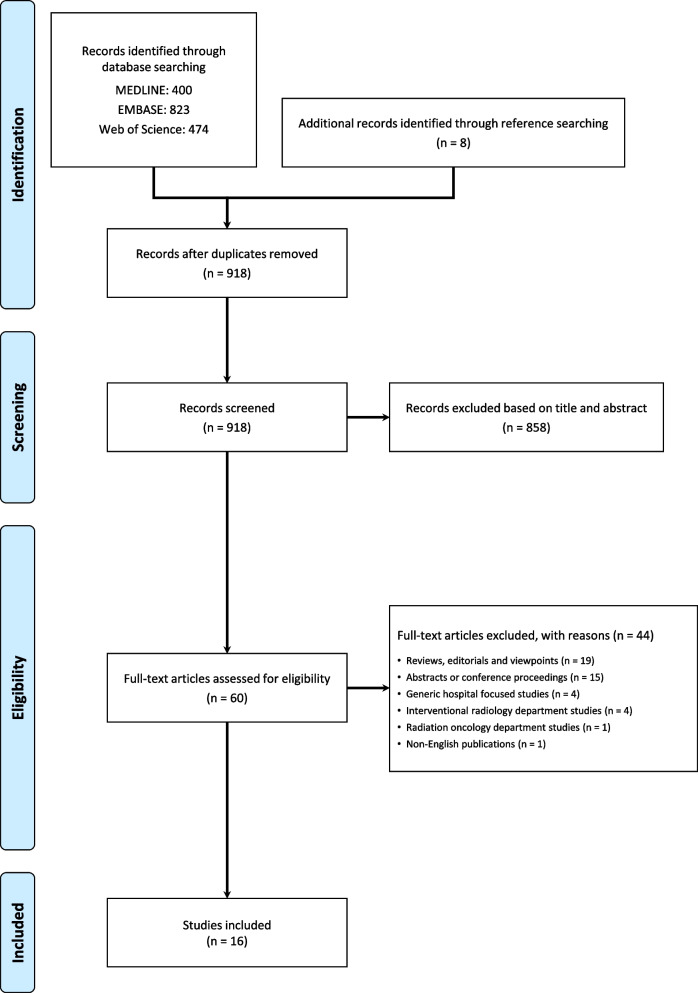
Flowchart depicting the study selection process, according to the Preferred Reporting Item for Systematic Reviews and Meta-Analyses (PRISMA)

Finally, a total of 16 articles which matched the inclusion criteria were eligible for narrative synthesis. A flowchart of study selection is shown in Fig. 1.

### Characteristics of the included publications

Baseline characteristics of articles are summarized in Table 1. Full database is published in Zenodo repository for data sharing (10.5281/zenodo.10473106). Two descriptive surveys were among the included publications [23, 24]. The remaining articles reported the results of studies on carbon footprint, consumption, and energy savings in diagnostic radiology departments using various quantitative descriptive methods. Seven articles originated from the USA, three from Switzerland, and two from the UK and also included contributions from Ireland, Canada, Australia, and Germany.

**Table 1:** This table provides a brief overview of the included studies, their sustainable actions, and principal results

Study	Country	Topic	GHG Protocol Corporate Standard scopes	Setting	Devices	Aim/objective	Sustainable actions	Principal results
Woolen (2023) [25]	USA	Energy consumption	Scope 2	Radiology imaging service	MRI scanners	Consumption and potential energy saving	Turning off MRI scanners during idle periods	Projected annual energy consumption per scanner: 82,700–171,100 kWh Nonproductive energy: 72–91% of total consumption
Gendy (2022) [24]	UK	Carbon footprint	Scope 1, Scope 2	Radiology imaging service	N/a	Current attitudes to environmental sustainably in radiology	Reducing travel, energy-saving measures	92% of survey participants showed high concern about climate crisis
Brown (2022) [26]	Canada	Energy consumption	Scope 2	Radiology imaging service	CT scanners	Consumption and potential energy saving	Turn off CT scanners in nonoperational hours	Potential energy saving for 1 CT scanner: 14,180 kWh per year Nonproductive energy: 40–80% of total consumption
McAlister (2022) [27]	Australia	Life-cycle assessment	Scope 2, Scope 3	Radiology imaging service	Ultrasound, x-ray, CT and MRI scanners	Greenhouse gas emissions and direct and indirect energy consumption	Reducing unnecessary imaging, choose lower energy imaging exams, turning off devices during idle periods	CO_2_ emissions were 17.5 kg/scan for MRI, 9.2 kg/scan for CT, 0.8 kg/scan for x-ray, and 0.5 kg/scan for US
Peters (2021) [23]	UK	Carbon footprint	Scope 1	Radiology training	N/a	Environmental impact of radiology trainees travel	Reducing travel, teleconferencing, and distance learning	Total emissions by radiology trainees: 122.5 tonnes of CO_2_
Büttner (2021) [18]	Germany	Energy consumption	Scope 2	Radiology imaging service	Monitors and workstations in radiology department	Consumption and potential energy saving	Energy-saving plan with automated shutdown/restart of workstations during idle periods	Potential energy consumption saving: 35,970 kWh (22.2 tons of CO_2_ and 14,388.28 USD/year)
Hainc (2020) [13]	Switzerland	Energy consumption	Scope 2	Radiology imaging service	Monitors and workstations in Radiology department	Consumption and potential energy saving	Turning off devices during idle periods	23,692 kWh potential energy saving per year, 45% of the initial energy consumption
Heye (2020) [14]	Switzerland	Energy consumption	Scope 2	Radiology imaging service	CT and MRI Scanners	Consumption and potential energy saving	Energy and cost-saving during idle and system-off states can be converted to more energy-efficient operating modes	Energy consumption imaging 614,825 kWh per year CT idle period: 78% (42 867 kWh) MRI idle period: 5.5–13.4% (8,177–16 038 kWh)
Alshqaqeeq (2020) [28]	USA	Energy consumption	Scope 2	Radiology imaging service	Ultrasound, x-ray, CT and MRI Scanners	Appropriateness of imaging exams related with energy consumption	Choose lower energy imaging exams	Potential US healthcare improvement: 24–240 million kWh per year (US $2.5–$25 million dollars per year)
Brodbeck (2019) [29]	Switzerland	Energy consumption	Scope 2	Radiology imaging service	N/a	Energy consumption	Development of informatic tools	Inform energy reduction strategies and improve scan protocols
Esmaeili (2018) [30]	USA	Life-cycle assessment	Scope 2, Scope 3	Radiology imaging service	MRI imaging services (MRI scanners and MRI room devices)	Greenhouse gas emissions and direct and indirect energy consumption	N/a	MRI scanner life-cycle energy: 104 kWh per patient 28% inhospital energy consumption (direct) 72% out-hospital energy consumption (indirect)
Martin (2018) [31]	USA	Life-cycle assessment	Scope 2, Scope 3	Radiology imaging service	Ultrasound, CT, and MRI scanners	Greenhouse gas emissions and direct and indirect energy consumption	N/a	Ultrasound has the least environmental impact compared to CT and MRI
Esmaeili (2015) [32]	USA	Life-cycle assessment	Scope 2, Scope 3	Radiology imaging service	CT imaging services (CT scanners and CT room devices)	Carbon footprint of CT scans by quantifying inhospital and out-of-hospital energy use	Expanding radiologists’ knowledge of unseen energy impacts of CT scans	CT scan life-cycle energy: 24–34 kWh per scan 25% inhospital energy consumption (direct) 75% out-hospital energy consumption (indirect)
McCarthy (2014) [33]	Ireland	Energy consumption	Scope 2	Radiology imaging service	Monitors and workstations in radiology department	Consumption and potential energy saving	Turning off computers, air-conditioning units, enabling sleep mode	116.304 kWh potential energy saving per year US $11,629 potential cost-saving per year 15 metric tons of CO_2_ emissions per year
Esmaeili (2011) [34]	USA	Energy consumption	Scope 2	Radiology imaging service	CT imaging services (CT scanners and CT room devices)	Energy consumed per month by CT machine in different states	Energy savings during CT idle period	Potential energy savings: 2,065 KWh per month (88% of the total)
Prasanna (2011) [35]	USA	Energy consumption	Scope 2	Radiology imaging service	Monitors and workstations in radiology department	Consumption and potential energy saving	Turn off devices at the end of workday and on weekends	Potential energy and cost savings: 76.31% (83,866.6 kWh and US $9,225.33, respectively)

### Topics, settings and devices

The main topics of articles related to environmental sustainability are as follows:

- Energy consumption and nonproductive periods of radiology devices (10/16, 62.5%)
- Life-cycle assessment (4/16, 25.0%)
- Carbon footprint and environmental impact (2/16, 12.5%)

In the field of diagnostic radiology, inhospital services are the main investigation’s setting (14/16, 87.5%), followed by radiology training areas (2/16, 12.5%). Diagnostic radiology devices were considered in 13 studies [13, 14, 18, 25–28, 30–35]. In particular, the devices whose energy consumption or environmental impact has been most studied are CT scanners (7/16, 43.7%), followed by MRI scanners (6/16, 37.5%), monitors and workstations (4/16, 25.0%), and ultrasound scanners (3/16, 18.7%).

**Fig. 2:**
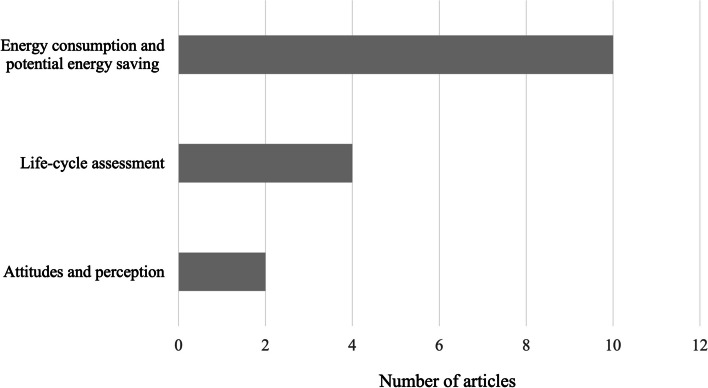
Number of articles grouped by topic of environmental impact

In summary, as shown in Fig. 2, the main issues on which studies in the literature have focused mainly concern the electricity consumption of diagnostic radiology equipment during the period of operation. Only a small proportion of them evaluate the entire life cycle of the equipment.

### Energy consumption and potential energy saving

A total of 11 studies [13, 14, 18, 25–29, 33–35] focused on energy consumption in radiology departments and identified potential areas for improvement. In 8 of these 11 studies [13, 14, 18, 25, 26, 33–35], the authors used real-time meters electricity/power consumption, as a tool of measuring the electricity consumed. Alshqaqeeq et al. [28] evaluated the appropriateness of imaging examinations in relation to energy consumption described for each type of imaging exam. Moreover, the unit of measurement adopted to describe electricity consumed is kilowatt-hour (kWh) in 9 out of 11 articles [13, 14, 18, 25, 26, 28, 33–35]. In addition to electrical energy expressed in kWh, some authors use carbon dioxide equivalent (CO_2_-eq), as scope 2 emissions, to describe environmental impact (3/11) [18, 25, 27, 33] and cost (US dollars) to describe economic impact of radiology service’s activity (7/11) [14, 18, 25, 28, 29, 33, 35].

In all studies about energy consumption, it was observed that the energy consumption of diagnostic radiology devices, such as MRI scanners and CT scanners, constituted a significant portion of the overall energy usage. Turning off devices during idle periods was highlighted as a sustainable action. Studies reported that a considerable percentage (ranging from 40 to 91%) [13, 14, 25–27, 34, 35] of the energy consumption by these devices was defined as non-productive, emphasizing the need for optimizing device usage and reducing idle times.

Sustainable actions aimed at energy saving and cost reduction were explored in the literature [13, 14, 18, 25–29, 33–35]. Turning off devices during idle period strategies were reported in 9/16 (56.2%) articles [13, 14, 18, 25–27, 33–35], and implementing informatic tools for workflow in 2/16 (12.2%) articles [14, 29] and optimizing operating modes in 2/16 (12.2%) articles [27, 28] were effective approaches. Significant potential energy savings were reported in 8/16 (50%) articles, with studies estimating annual savings ranging from thousands to tens of thousands of kWh (ranging from 14,180 to 171,000 kWh) [13, 14, 18, 25, 26, 33–35]. Cost savings were also identified in 7/16 (43.7%) articles [14, 18, 25, 28, 29, 33, 35], emphasizing the economic benefits of energy-efficient practices applied to the analyzed radiology devices.

### Life-cycle assessment

Four studies [27, 30–32] conducted life-cycle assessments to evaluate the overall energy consumption and environmental impact associated with radiology services. These assessments considered both direct energy consumption within the hospital setting and indirect energy consumption associated with the manufacturing, transportation, and disposal of imaging devices. In these four studies, a transparent and detailed life-cycle assessments approach not only is used to assess the direct and indirect energy impact of radiological devices but also frequently includes the evaluation of public health implications. Among medical imaging modalities, ultrasounds are found to have the least environmental impact, significantly different from other modalities. For MRI, the carbon footprint associated with the entire individual service has been measured up to a maximum of 22.4 kg of CO_2_-eq, as Scope 2 and Scope 3 emissions. This value includes both inhospital process energy (29 kWh per patient) and off-hospital energy (about 75 kWh per patient), required not only for electricity generation during its medical use but also for the manufacturing of the MRI scanner itself and disposable and reusable products used during diagnostic activities. Only approximately 28% of the total MRI life-cycle energy is used for image acquisition, suggesting potential improvements to reduce the environmental impact [27, 30]. However, there is relatively less attention given to the deactivation and disposal of radiological equipment. These analyses provide a basis for comparing environmental impacts across different imaging modalities throughout their entire life cycle, with implications for cost-effectiveness analyses and the development of sustainable policies in the medical field.

### Attitudes and perception

Two studies [23, 24] provided insights into the attitudes and perceptions of radiology staff toward environmental sustainability. In both studies, the survey method was used. In the first study [23], the environmental impact of radiology students’ travel during their internship was assessed. Based on the responses, the environmental impact of the students’ travel was calculated (the total emissions released by all radiology trainees would be 122.5 tons of CO_2_, equating to 73.8 return flights from London to New York), and suggestions were made to improve their environmental sustainability. For example, trainers and trainees were happy to use e-mail or video links, rather than face to face during the training, to reduce their environmental impact. In the second study [24], an online survey was conducted among radiology staff to assess their attitudes towards the climate emergency. The results showed a high level of concern regarding this issue. Active commuting represents only a small percentage of the travel related to radiology services. Energy-saving measures are commonly implemented in radiology departments, but they constitute only a fraction of the overall energy usage.

These two studies have shown that there is a common sensitivity among radiology staff regarding the environmental impact topic. Further surveys, including international ones, would be desirable to gain a broader understanding of the perception of the theme of energy sustainability. However, there is already significant potential to reduce the carbon footprint of radiology services by minimizing travel, both for work and training in radiology.

### Quality appraisal

QualSyst total score ranged from 50.0 [29] to 100.0% [14, 25–27] (median 90%, interquartile range 80–95%) with 14/16 (87.5%) articles [13, 14, 18, 23–28, 30, 32–35] over or equal to the 80% of high-quality level. The included studies performed poorly on the quality of data collection and analysis and on the use of verification measures in sufficient detail. Table 2 represents the full results of QualSyst quality assessment of included publications.

**Table 2:** This table provides full results of QualSyst quality assessment of included publications

		Study														
Criteria	Prasanna (2011) [35]	Esmaeili (2011) [34]	McCarthy (2014) [33]	Esmaeili (2015) [32]	Martin (2018) [31]	Esmaeili (2018) [30]	Brodbeck (2019) [29]	Alshqaqeeq (2020) [28]	Heye (2020) [14]	Hainc (2020) [13]	Büttner (2021) [18]	Peters (2021) [23]	McAlister (2022) [27]	Brown (2022) [26]	Gendy (2022) [24]	Woolen (2023) [25]
Question/objective sufficiently described?	2	2	2	2	2	2	1	2	2	2	2	2	2	2	2	2
Study design evident and appropriate?	2	2	2	2	2	2	1	2	2	2	1	2	2	2	2	2
Context for the study clear?	2	2	2	2	1	2	1	2	2	2	2	2	2	2	2	2
Connection to a theoretical framework/wider body of knowledge?	2	2	2	2	2	2	1	2	2	2	2	2	2	2	2	2
Sampling strategy described, relevant, and justified?	2	2	2	2	2	2	1	2	2	2	1	2	2	2	2	2
Data collection methods clearly described and systematic?	2	1	1	1	1	1	0	2	2	2	2	2	2	2	2	2
Data analysis clearly described and systematic?	2	1	1	1	1	1	1	2	2	2	2	2	2	2	2	2
Use of verification procedure(s) to establish credibility?	1	1	1	1	1	1	1	1	2	1	1	1	2	2	1	2
Conclusions supported by the results?	1	1	2	2	1	2	1	1	2	2	2	2	2	2	2	2
Reflexivity of the account?	2	2	2	1	1	1	2	2	2	2	2	2	2	2	1	2
**Total (0–20)**	**18 (90%)**	**16 (80%)**	**17 (85%)**	**16 (80%)**	**14 (70%)**	**16 (80%)**	**10 (50%)**	**18 (90%)**	**20 (100%)**	**19 (95%)**	**17 (85%)**	**19 (95%)**	**20 (100%)**	**20 (100%)**	**18 (90%)**	**20 (100%)**

## Discussion

This systematic review provides a comprehensive overview of various studies that explore crucial aspects such as energy consumption patterns, carbon footprint, and sustainable initiatives implemented within radiology departments in diverse countries. The key outcomes from these studies collectively illuminate the current state of environmental practices in radiology departments and may contribute valuable insights to explore and promote eco-friendly approaches in the field.

One prominent finding was the high-energy consumption associated with medical devices installed in radiology departments. The studies highlighted the need for optimizing device usage and reducing idle periods to address the nonproductive energy consumption, which accounted for a substantial portion of the overall energy usage. This nonproductive energy consumption accounted for a valuable proportion of the overall energy usage, ranging from 40 to 91% in the studies [13, 14, 25–27, 34, 35]. Studies taking into consideration shutting down MRI and CT units during idle periods [14, 25–27, 30–32, 34] have shown that it is possible to make radiology departments more energy efficient, showing significant benefits in terms of environmental sustainability, with energy savings ranging from 14,180 to 171,000 kWh per year and annual cost savings ranging from US $9,225 to 14,328 per device. The energy consumption of the reporting workstations is also not negligible and can be achieved through simple changes in device configuration, enabling for example automatic shutdown after long periods of inactivity [13, 18, 33, 35]. Other aspects reported by Woolen et al. [36] include strategies for reducing consumables, waste, heating and cooling equipment, and emissions from equipment disposal and replacement.

Additionally, four studies highlighted the importance of considering the overall life-cycle energy consumption of diagnostic units [27, 30–32]. This includes not only the energy consumed during their operational phases but also the energy expended during the manufacturing, transportation, and disposal stages, providing insights into potential areas for improvement. An unexplored topic in environmental impact in the life cycle of radiological equipment concerns their obsolescence and renewal [37, 38], in which the need for planned equipment renewal is stated to avoid failures and delays in diagnosis and patient care. Radiology devices are composed of plastics, metals, and rare-earth elements that, if not properly disposed of, can cause environmental pollution [37].

Another result was the growing concern among radiology staff regarding the climate change. Survey-based studies revealed a high level of awareness and a willingness to engage in sustainable practices. The findings highlighted the importance of promoting environmentally conscious behaviors, such as reducing travel, implementing energy-saving measures, and embracing teleconferencing and distance learning options [23, 24]. These actions not only contribute to carbon footprint reduction but also align with the broader sustainability goals of healthcare systems. For the future, there is a need to foster and promote the environmental culture even more in radiological staff healthcare workers.

The dissemination of training courses, learning groups, teleconferencing, and remote consultations must be promoted and implemented within radiology services. The goal must be to reduce the ~ 1.85 tons of CO_2_, calculated by Peters et al. [23], caused by the travel of radiology staff for work and training. While online participation in health conferences or training courses may present challenges related to the perceived quality of education by learners and potential disparities in user experience due to factors such as Internet bandwidth and real-time or synchronous delivery, a thorough evaluation of the pros and cons is imperative for both environmental sustainability and the quality of ongoing education. However, findings by Vaona et al. [39] suggest that, when compared to traditional learning, e-learning may have little or no impact on patient outcomes or health professionals’ behaviors, skills, or knowledge. While e-learning could be more effective in specific medical education settings, overarching claims of its inherent superiority over traditional learning may be misleading.

Two studies [14, 29] highlighted the importance of informatics tools for analyzing workflow and energy consumption. These tools offer valuable data to inform energy reduction strategies and optimize scan protocols, leading to more energy-efficient practices in radiology departments. In addition, these tools allow to reduce energy consumption considering an organizational approach (*e.g.*, turning off equipment when there is no work activity) without evaluating an engineering approach, which has been used in other published work in the healthcare field [4, 5].

On the topic of carbon emissions, there is limited analysis of the total data. No specific article identifies the type of carbon emission according to the GHG Protocol Corporate Standard [8]. When discussing space heating or cooling, vehicle travel by healthcare professionals is considered within the Scope 1 category of direct GHG emissions. On the other hand, emissions from energy consumption or the production of equipment fall under Scope 2 and Scope 3, representing electricity or other indirect GHG emissions.

Proper classification of emissions in the radiology area is important because in some cases a diagnostic activity does not always necessarily correspond to a carbon emission. For example, if the energy used to operate CT and MRI scanners was derived from production through photovoltaic panels, the carbon emissions would be close to zero.

The other side of the coin on the topic of sustainability in radiology concerns the effective management of waste generated during clinical activities [17]. These wastes can result in significant carbon emissions and financial burdens for radiology departments [40]. For example, residual and unwanted effects of iodinated and gadolinium-based contrast agents are often disposed of in wastewater systems or clinical waste streams, contributing to increased environmental pollution. However, there are virtuous examples where these contrast agents are collected and recovered, as demonstrated by initiatives such as the GREENWATER study [41] and other experiences [40, 42]. This study aims to assess the actual amounts of iodinated and gadolinium-based contrast agents recoverable from patient urine collected after CT and MRI examinations.

Different reviews on the topic of energy conservation in diagnostic radiology can be found in the literature, but none of them presents a systematic approach [10, 36, 43]. In the field of interventional radiology [19, 44], where studies related to environmental sustainability in the operating room were also considered, the same issues of equipment energy consumption, carbon footprint associated with medical activities, and staff awareness in environmental sustainability were identified. Waste generation and recycling of material used during interventional procedures were also added to these topics. Both, although not identified in the selected articles in our literature review, could also be associated with diagnostic radiology activities. Shum et al. [19] concluded that there is a need to discuss environmental awareness in their day-to-day conversation and actively contribute to the global green movement by delivering practical actions to clinical practice. In the literature review related to environmental sustainability in radiation oncology [20], the recurring themes are always carbon footprint and electricity consumption, thus demonstrating how these two themes are the most frequent throughout the literature related to the macro area of radiology. Bloom et al. [20] identify that there are several gaps in the literature which were identified including comparison of various treatment modalities such as single *versus* multifraction treatment and photon *versus* proton *versus* carbon therapy energy requirements.

### Study limitations

Despite the valuable insights provided by the included studies, this systematic review has some limitations that should be taken into consideration. Firstly, the scope of this review is limited to the studies available within the selected timeframe and databases. Although efforts were made to include a wide range of articles, it is possible that some relevant studies were inadvertently excluded, even considering those publications that are not published in English language. Secondly, the generalizability of the findings of the included studies may be limited due to the variability in study designs, settings, and geographic locations. The included studies were conducted in different countries, with variations in healthcare systems, infrastructure, and energy sources. These contextual factors can influence energy consumption patterns and sustainability practices in radiology departments, making it challenging to draw universal conclusions. Furthermore, the heterogeneity of the data collected across studies poses a challenge for direct comparisons and synthesis. Variations in the unit of measurement, data collection methods, and outcome reporting may impact the precision and consistency of the results. The differences in measurement units (*e.g.*, kWh, kg CO_2_-eq, US dollar per kWh) and data collection methods (*e.g.*, real-time metered electricity consumption, survey responses) may introduce some degree of uncertainty and make it challenging to directly compare the findings. It is also important to acknowledge the potential presence of publication bias in systematic reviews. Studies with statistically significant or positive results are more likely to be published, while studies with null or negative findings may be underrepresented [45]. This bias may affect the overall conclusions drawn from the included studies. Lastly, the review relies on the quality and rigor of the included studies. While efforts were made to ensure the inclusion of high-quality studies, variations in study design, sample size, and methodology may influence the reliability and validity of the findings. Through the QualSyst tool, the overall quality can be considered as satisfactory, with a median article quality equal to 90% (interquartile range 80–95%).

## Conclusions

In conclusion, this systematic review emphasizes the importance of environmental sustainability in radiology departments and the potential for energy savings, carbon footprint reduction, and cost optimization through sustainable actions like the following:

- Turning off devices during idle period helps to save energy and consequently reduce emissions and costs.
- Implementing power management informatic systems that automatically turn off or reduce the power consumption of idle equipment when not in use
- Investing in teleradiology and promoting remote collaboration and consultations can lead to lower carbon emissions.
- Favoring the most energy-efficient diagnostic technique and energy-efficient imaging devices in relation to the clinical question, without compromising the quality of service.
- Educating radiological staff regarding energy-saving practices and sustainability goals.

The findings provide valuable insights for healthcare providers, policymakers, and researchers to develop strategies and initiatives aimed at promoting environmental sustainability in radiology practice. By implementing these measures, radiology departments can contribute to a greener and more sustainable healthcare system, aligning with global sustainability goals and ensuring the well-being of both patients and the planet. In the future, it will be necessary to prospectively evaluate the introduction of sustainable actions in entire radiology departments, considering the life cycles of radiology equipment with a focus on the costs and environmental impact of aging and refurbishing units. In the next future, a radiology department could also be evaluated for its quantified level of environmental sustainability, opening a virtuous competition among hospitals and centers.

### Supplementary Information

**Additional file 1. Supplementary file 1. Supplementary Methods. **Literature search strategy. Full search strings.

## Data Availability

Full database is published in Zenodo repository for data sharing. See: Roletto A, Zanardo M, Bonfitto GR, Catania D, Sardanelli F, Zanoni S. (2024). The environmental impact of energy consumption and carbon emissions in radiology departments: a systematic review [Data set]. Zenodo. 10.5281/zenodo.10473106.

## Abbreviations

CO_2_-eq: Carbon dioxide equivalent
CT: Computed tomography
GHG: Greenhouse gas
MRI: Magnetic resonance imaging
kWh: Kilowatt-hour
