# Supplementary material for: The environmental impact of energy consumption and carbon emissions in radiology departments: a systematic review
Source: Eur Radiol Exp. 2024 Feb 29;8:35. doi: 10.1186/s41747-024-00424-6 (PMC10902235; doi:10.1186/s41747-024-00424-6)
Supplement: Supplementary file 1 — Additional file 1. Supplementary file 1. Supplementary Methods. Literature search strategy. Full search strings. [file 41747_2024_424_MOESM1_ESM.docx]

## The environmental impact of energy consumption and carbon emissions in radiology departments: a systematic review

## ELECTRONIC SUPPLEMENTARY MATERIAL

## Supplementary Methods

### Literature search strategy

Update: August 30^th^, 2023

Databases used: MEDLINE (PubMed), EMBASE (Elsevier) and Web of Science (Clarivate Analytics).

A controlled vocabulary (medical subject headings in PubMed and EMBASE thesaurus keywords in EMBASE) was used. The search strings were built using the following strategy, based on the PICO model:

| 'environmental sustainability'/exp + synonyms |
| --- |
| 'radiology'/exp + synonyms OR 'radiology department'/exp |
| 'energy consumption'/exp + synonyms OR 'carbon emission'/exp OR 'recycling'/exp OR 'waste'/exp |

### Full search strings

The full search string for **EMBASE (Elsevier)** was:

('environmental sustainability'/exp OR 'environmental sustainability' OR 'sustainability, environmental' OR 'energy consumption'/exp OR 'energy consumption' OR 'sustainability'/exp OR 'waste'/exp OR 'garbage' OR 'residue' OR 'waste' OR 'waste products' OR 'recycling'/exp OR 'equipment reuse' OR 'recycling' OR 'recycling, waste' OR 'resource recovery' OR 'utilization, waste' OR 'waste recycling' OR 'waste utilization' OR 'environment friendly' OR 'environment impact' OR 'climate change'/exp OR 'climate change' OR 'climate sensitivity' OR 'climate variability' OR 'climatic change' OR 'greenhouse effect'/exp OR 'effect, greenhouse' OR 'global warming' OR 'greenhouse effect' OR 'warming, global' OR 'carbon emission'/exp OR 'carbon emission' OR 'carbon emissions' OR 'carbon footprint'/exp OR 'ghg footprint' OR 'carbon footprint' OR 'greenhouse gas footprint' OR 'total ghg emission' OR 'total carbon emission' OR 'total green house gas emission' OR 'total greenhouse gas emission') AND ('radiology'/exp OR 'radiologic investigation' OR 'radiologic technology' OR 'radiological investigation' OR 'radiological technology' OR 'radiology' OR 'roentgenologic investigation' OR 'roentgenological investigation' OR 'roentgenology' OR 'rontgenology' OR 'technology, radiologic' OR 'radiology department'/exp OR 'x ray department' OR 'hospital radiology department' OR 'radiodiagnosis department' OR 'radiography department' OR 'radiological department' OR 'radiology department' OR 'radiology department, hospital' OR 'radiology service' OR 'roentgen department' OR 'roentgen facility') AND ('energy'/exp OR 'energy' OR 'energy dependence' OR 'green initiatives' OR green)

The full search string for **PubMed (MEDLINE)** was:

# 1 TOPIC: (Environmental sustainability)

(“Environmental sustainability”[MeSH Terms] OR “Sustainable Development” OR “Sustainable Development Goals” OR “Environmental Policies” OR “Environmental Policy” OR “Policies, Environmental” OR “Policy, Environmental” OR “Environmental Impact” OR “Environmental Impacts” OR “Impact, Environmental” OR “Impacts, Environmental” OR “Environmental Health” OR “Environmental Health Science” OR “Health, Environmental”)

# 2 TOPIC: (Radiology department)

(“Radiology”[MeSH Terms] OR “Radiology”) AND (“Diagnostic Imaging”[MeSH Terms] OR “Diagnostic Imaging” OR “Imaging, Diagnostic” OR “Imaging, Medical” OR “Medical Imaging”) (“Radiography”[MeSH Terms] OR “X-ray” OR “X-ray diagnosis” OR “X-ray image” OR “diagnostic imaging” OR “echography” OR “echotomography” OR “radiography” OR “radioisotope scanning” OR “radionuclide imaging” OR “roentgenography” OR “ultrasonic diagnosis” OR “ultrasonography” OR “ultrasound”)

# 3 TOPIC: (Energy consumption) AND (Carbon emission) AND (Recycling) AND (Waste)

(“Energy consumption”[MeSH Terms] OR “Renewable Energy” OR “Sustainable Energy” OR “Conservation of Energy Resources” OR “Energy Resources Conservation” AND “Carbon Footprint”[MeSH Terms] AND “Recycling”[MeSH Terms] AND “Waste”[MeSH Terms])

The full search string for **Web of Science (Clarivate Analytics)** was:

Radiology department (All Fields) or Radiology (All Fields) and Environmental sustainability (All Fields) and energy consumption (All Fields) and Carbon emission (All Fields) and Environmental impact (All Fields) and Recycling (All Fields) and Waste (All Fields) and Article OR Review (Document Type) and Radiology Nuclear Medicine Medical Imaging (Web of Science Categories)
